# Novel Repurposing of Empagliflozin-Loaded Buccal Composite (Chitosan/Silk Fibroin/Poly(lactic acid)) Nanofibers for Alzheimer’s Disease Management via Modulation of Aβ–AGER–p-tau Pathway

**DOI:** 10.3390/pharmaceutics18010083

**Published:** 2026-01-08

**Authors:** Walaa A. El-Dakroury, Samar A. Salim, Abdelrahman R. Said, Gihan F. Asaad, Mohamed F. Abdelhameed, Marwa E. Shabana, Mohamed M. Ibrahim, Sara G. Abualmajd, Haidy H. Mosaad, Aliaa A. Salama, Shrouk E. Asran, Mayar L. Amer, Ahmed S. Doghish, Fatma Sa’eed El-Tokhy

**Affiliations:** 1 Department of Pharmaceutics and Industrial Pharmacy, Faculty of Pharmacy, Badr University in Cairo (BUC), Badr City 11829, Cairo, Egypt; walaa.ahmed2@buc.edu.eg (W.A.E.-D.); abdelrahman.ragab@buc.edu.eg (A.R.S.); fatma.saeed@buc.edu.eg (F.S.E.-T.); 2 Nanotechnology Research Center (NTRC), The British University in Egypt (BUE), El-Sherouk City 11837, Cairo, Egypt; 3 Department of Pharmacology, Medical Research and Clinical Studies Institute, National Research Centre, Dokki, Giza 12622, Cairo, Egypt; dr_g.asaad@yahoo.com (G.F.A.); fayed.nrc@gmail.com (M.F.A.); 4 Pathology Department, National Research Centre, Dokki, Giza 12622, Cairo, Egypt; marwashabana999@gmail.com; 5 Faculty of Pharmacy, Badr University in Cairo (BUC), Badr City 11829, Cairo, Egypt; mohamedmahmoud8942@gmail.com (M.M.I.); saragamal3456@gmail.com (S.G.A.); haidyhamdy080@gmail.com (H.H.M.); alyalya672@gmail.com (A.A.S.); shookemad161@gmail.com (S.E.A.); mayaramer01@gmail.com (M.L.A.); 6 Biochemistry and Molecular Biology Department, Faculty of Pharmacy (Boys), Al-Azhar University, Nasr City 11231, Cairo, Egypt; 7 Department of Biochemistry, Faculty of Pharmacy, Badr University in Cairo (BUC), Badr City 11829, Cairo, Egypt

**Keywords:** Alzheimer’s disease, empagliflozin, fibroin, chitosan, polylactic acid, nanofibers

## Abstract

**Background/Objectives**: Empagliflozin (EMPA) was repurposed for Alzheimer’s disease (AD) treatment via buccal delivery, exploiting novel nanofibers (NFs) integrating chitosan (Cs), silk fibroin (Fb), and poly(lactic acid) (PLA). **Methods**: EMPA-loaded Cs/Fb/PLA NFs were electrospun in different formulations to optimize the formulation parameters. The optimized formulation was then investigated for its enhanced in vivo effect. **Results**: Optimized nanofiber diameters ranged from 459 ± 173 to 668 ± 148 nm, possessing bead-free morphology confirmed by SEM and satisfactory mechanical properties. EMPA was successfully well-dispersed in the polymer matrix as evidenced by FTIR, XRD, and drug content. The optimized NFs displayed a hydrophilic surface (contact angle < 90°), and biphasic drug release with sustained EMPA liberation (84.98% over 24 h). In vivo, buccal EMPA-Cs/Fb/PLA NFs in an AlCl_3_-induced AD rat model significantly reduced brain-amyloid-β, phosphorylated tau, IL-1β, and AGER expression by 2.88-, 2.64-, 2.87-, and 2.50-fold, respectively, compared to positive controls, and improved locomotor activity (1.86-fold) and cognitive performance (T-maze) (4.17-fold). Compared to pure EMPA, the nanofiber formulation achieved further reductions in amyloid-β (1.78-fold), p-tau (1.42-fold), IL-1β (1.89-fold), and AGER (1.38-fold), with efficacy comparable to memantine. Histopathological examination revealed preservation of the hippocampal neuronal structure. **Conclusions**: The findings suggest EMPA-loaded Cs/Fb/PLA NFs as a promising non-invasive, sustained-release buccal delivery platform for AD therapy, offering multimodal neuroprotection through modulation of the Aβ–AGER–p-tau axis.

## 1. Introduction

Alzheimer’s disease (AD), the main cause of dementia worldwide, is a progressive neurological illness that mostly affects older people. AD causes cognitive decline, notably memory loss. First, people may have trouble remembering recent events or conversations, which can lead to language, reasoning, and decision-making issues. As the illness advances, people may struggle to manage daily tasks, change their attitude and behavior, and need significant help with basic daily tasks. Due to neuronal cell damage, notably in memory retention and advanced cognitive functions, several symptoms occur [1,2].

AD affects people worldwide and poses economic issues. AD costs over $1 trillion annually, including medical costs, care, and social impacts. Recent findings suggest that early detection, public health initiatives, and awareness have reduced AD burden in certain places [3].

The existing pharmacological treatments for AD provide only modest advantages [4]. The cornerstone of treatment comprises five FDA-approved drugs: donepezil, galantamine, rivastigmine, memantine, and a combination of memantine with donepezil. These drugs increase brain neurotransmitters, improving nerve cell transmission. These medications only temporarily relieve symptoms and do not address disease causes or slow neurodegeneration [2]. These therapies for AD, including cholinesterase inhibitors and memantine, primarily provide short-term symptom relief but fail to modify the underlying disease process, which continues to progress unabated [5]. These drugs often lose effectiveness after 6 to 12 months and show variable results across patients [6]. Side effects such as gastrointestinal issues reduce adherence, and newer treatments like monoclonal antibodies (e.g., aducanumab, lecanemab) pose risks of brain swelling and require ongoing infusions, which are costly and inconvenient [7]. Current diagnostics frequently reveal AD at moderate or advanced stages, limiting early intervention, and the disease’s complexity makes a single therapy option unlikely to work for all people [8].

In this context, drug repurposing presents a promising strategy, particularly the use of empagliflozin (EMPA), a sodium-glucose co-transporter 2 (SGLT2) inhibitor originally developed for type 2 diabetes (T2DM). EMPA has garnered attention as a potential therapeutic strategy for AD, driven by its pleiotropic mechanisms that intersect with AD pathophysiology. Central to its proposed efficacy is its ability to ameliorate insulin resistance, a hallmark of AD often termed “type 3 diabetes” [9]. EMPA enhances peripheral insulin sensitivity and glucose metabolism, which may restore cerebral insulin signaling, thereby improving synaptic plasticity and reducing the hyperphosphorylation of tau proteins [10]. Additionally, EMPA exhibits anti-inflammatory properties by suppressing pro-inflammatory cytokines such as interleukin-6 (IL-6) and tumor necrosis factor-alpha (TNF-α) via inhibition of the NLRP3 inflammasome, a pathway implicated in neuroinflammation and neurodegeneration [11]. Oxidative stress, another critical AD mechanism, is mitigated through empagliflozin’s upregulation of antioxidant enzymes like superoxide dismutase (SOD) and reduction in reactive oxygen species (ROS) production, thereby protecting neurons from mitochondrial dysfunction [12].

Furthermore, preclinical studies suggest that empagliflozin may attenuate amyloid-beta (Aβ) aggregation by modulating advanced glycation end products (AGEs) and receptor for AGE (AGER) interactions, which are known to exacerbate Aβ deposition and neurotoxicity. Emerging evidence also highlights its role in promoting autophagy, a cellular clearance mechanism disrupted in AD, through AMP-activated protein kinase (AMPK) activation, facilitating the removal of misfolded proteins [13,14]. These findings demonstrate empagliflozin’s multimodal potential, which could lead to speedier and cheaper novel therapies, especially because its safety in diabetics is well-established. Its direct CNS penetration, long-term safety, and efficacy in non-diabetic AD patients need further study. However, its capacity to target numerous AD pathways makes EMPA an attractive medication-repurposing candidate for neurodegenerative therapies.

However, developing new, more effective medicines is challenging. Drug candidates must cross the blood–brain barrier and treat complex pathologies like amyloid plaques and tau tangles while protecting brain cells. Thus, many clinical trials have failed, forcing several major pharmaceutical companies to stop developing AD drugs due to difficulties and high costs. This underscores the need for innovative therapeutic options that can change the illness’s trajectory and holistic care frameworks to help patients and their families with AD [15]. Buccal drug delivery systems represent a significant advancement in the quest for effective therapies for AD, addressing critical challenges associated with conventional drug administration routes. The buccal mucosa’s rich vascular network and high permeability make it a unique systemic medication delivery route. It allows quick therapeutic drug absorption into the bloodstream via facial and jugular veins. Oral delivery’s main drawback is hepatic first-pass metabolism, which lowers medication bioavailability, especially for substances vulnerable to liver or gastrointestinal tract enzymatic degradation [16]. For AD therapeutics, which frequently include peptides, proteins, or small molecules requiring precise and sustained plasma concentrations, the buccal route enhances therapeutic efficacy by maintaining optimal drug levels while minimizing systemic toxicity [17].

Managing chronic neurodegenerative disorders like AD requires long-term patient compliance, and buccal administration is non-invasive. Recent mucoadhesive films, gels, and nanoformulations improve medication retention at the absorption site, lengthening contact duration and allowing controlled release [18]. These systems also circumvent the challenges of the blood–brain barrier (BBB) by leveraging systemic circulation to gradually distribute drugs into the brain, potentially enhancing the targeting of amyloid-beta plaques or tau proteins. Despite challenges such as mucosal permeability, saliva washout, and formulation stability, material science and pharmacokinetic modeling continue to improve buccal platforms for CNS applications [19]. Overall, buccal drug delivery systems could hold transformative potential for AD treatment, combining drug precision, patient-centric administration, and enhanced therapeutic outcomes in a field urgently needing innovative solutions.

Combining nanotechnology, especially nanofiber mats, with buccal drug delivery devices is a revolutionary way to treat AD. Electrospun nanofiber mats have a high surface area-to-volume ratio, boosting drug loading capacity and buccal mucosa contact, consequently improving drug absorption and bioavailability [20]. Their tunable polymeric composition, such as with poly(lactic-co-glycolic acid) (PLA) or chitosan (CS), allows precise modulation of drug release kinetics, facilitating sustained or stimuli-responsive delivery critical for maintaining therapeutic plasma concentrations of AD drugs like donepezil or rivastigmine [21,22,23]. Notably, nanofibers can encapsulate both hydrophilic (e.g., peptides targeting amyloid-beta plaques) and hydrophobic agents (e.g., curcumin, a neuroprotective antioxidant), overcoming formulation challenges associated with AD’s multifactorial pathology [23,24].

Nanofiber mats’ mucoadhesive qualities lengthen residence duration on the buccal mucosa, minimizing salivary washout and improving systemic absorption without hepatic metabolism for medicines with low oral bioavailability [25]. However, challenges such as scalability, reproducibility, and long-term mucosal biocompatibility require rigorous evaluation, as inflammatory responses to prolonged nanofiber exposure could counteract therapeutic benefits [26].

Poly (lactic acid) (PLA), a synthetic polymer, has been employed in regenerating both soft and hard tissues. Its rich history of successful applications has sparked considerable interest in its potential as a biomaterial [27]. According to prior research, PLA experiences degradation via the chemical hydrolysis of its unstable ester bonds, leading to the production of non-toxic and biocompatible lactic acid [28]. This material is distinguished by its exceptional mechanical flexibility, reduced antigenicity, and ease of processing. Nonetheless, its limitations include pronounced hydrophobicity, low water absorptivity, insufficient cell attachment, and a diminished cell growth rate [27]. A varied selection of natural polymers, including fibroin, chitosan, gelatin, hyaluronic acid, cellulose, and collagen, along with their derivatives, has been skillfully crafted into nanofibrous membranes designed to replicate the extracellular matrix of skin tissues [29].

Chitosan (Cs), a cationic natural polymer, is biodegradable, biocompatible, antimicrobial, cell adhesion, non-toxic, and chemically functionalizable [30,31,32]. It can be easily shaped into hydrogels and nanofibers [33,34]. Cs electrospun membranes promote cell adhesion and proliferation, making them desirable for wound healing [35]. Cs improves mucoadhesion of nanofibers due to its unique physico-chemical and biological features. As a cationic biopolymer, Cs strongly adheres to mucosal surfaces through electrostatic interactions, hydrogen bonding, and hydrophobic effects with negatively charged mucins [36,37]. Electrospun nanofiber systems like chitosan/polyethylene oxide blends increase mucoadhesion by enhancing fiber swelling and tissue contact [38]. Silk fibroin (Fb), a natural protein extracted from Bombyx mori silk, has emerged as a viable biomaterial for fabricating nanofiber-based systems in AD implications. Its biocompatibility, biodegradability, and superior mechanical qualities render it appropriate for neurological therapy. Recent studies have included Fb nanofibers in diagnostic platforms and therapeutic scaffolds for AD. Fb-based thin films have been utilized in electrochemical immunosensors to detect amyloid-β peptides, critical biomarkers in AD pathogenesis, exhibiting sensitive and specific diagnostic capabilities [39].

Moreover, protein-based electrospun nanofibrous scaffolds, such as Fb, have showed promise in therapeutic and regenerative approaches by acting as carriers for bioactive substances or facilitating stem cell distribution for neurological regeneration in dementia models [40]. Fibroin’s stable secondary structure allows the controlled and extended release of therapeutic medicines, including antioxidants and anti-amyloidogenic compounds [41]. Furthermore, its antioxidant properties protect brain cells from oxidative stress, which accelerates AD [41]. Fibroin nanofibers are a promising multifunctional platform for AD diagnosis, treatment, and neuroprotection due to their non-immunogenic profile and customizable pace. Hence, this study explores the repurposing of empagliflozin, a sodium-glucose co-transporter 2 (SGLT2) inhibitor traditionally used for type 2 diabetes, as a novel therapeutic candidate for AD. This research develops empagliflozin-loaded Cs/Fb/PLA nanofiber mats for buccal delivery to overcome the current methods’ limitations and provide sustained release, effectiveness, and targeted action. Combining buccal mucosal administration with nanofiber technology may synergistically improve drug efficacy and patient compliance, potentially revolutionizing AD management.

We hypothesize that incorporating the natural polysaccharide chitosan within silk fibroin/poly(lactic acid) nanofiber matrices will synergistically enhance mucoadhesion, biocompatibility, and the sustained release of empagliflozin, thereby improving its buccal absorption and neuroprotective efficacy against AD through modulation of the Aβ–AGER–p-tau signaling cascade.

## 2. Materials and Methods

Empagliflozin (EMPA) was kindly provided as a research gift by ATCO Pharma, Quesna, Egypt. Bombyx mori cocoons were supplied by Silk Valley for Development, Cairo, Egypt. Phosphate buffer salts were sourced from Advent ChemBio, Mumbai, India. Poly(lactic acid) (PLA) (Ingeo^TM^ 6202D) (M_W_: 239,000 g/mol as determined by gel permeation chromatography in dimethylformamide using a Shimadzu UFLC-20AD (Kyoto, Japan); relative viscosity 3.1; dispersity index (Mw/Mn) 2.07; L/D ratios of 24:1 to 30:1) was sourced from Nature Works LLC., Plymouth, MN, USA. Purified chitosans (Cs) 15 and 100 kDa (90% deacetylation) were sourced from Polysciences Inc. (Warrington, FL, USA). Formic acid was obtained from Sigma-Aldrich Chemical Company, St. Louis, MO, USA. Analytical-grade absolute ethanol and chloroform were procured from El-Nasr Pharmaceutical Chemicals Company, Cairo, Egypt. Additionally, sodium carbonate, calcium chloride, and calcium nitrate were purchased from Piochem, Giza, Egypt.

### 2.1. Animals

We obtained 48 male Wistar rats (150–180 g) from the National Research Centre animal house (Giza, Egypt). The rats were kept in plastic cages (12 h light/dark cycle, 22 °C temperature, and 65% relative humidity). They were administered food and water ad libitum following the guidelines outlined in the National Institutes of Health Handbook for the Care and Use of Laboratory Animals. The BUC ethical committee approved all experimental procedures (approval no. IACUC/PHA/169/A/2024).

### 2.2. Analytical Chemicals, ELISA Kits, and RT-PCR Kits

Aluminum chloride (Sigma-Aldrich Company, St. Louis, MO, USA). Rat amyloid beta peptide 1-42 (Aβ1-42 ELISA Kit; Biorbyt LLC, San Francisco, CA, USA); (Catalog # orb410690). Rat phospho-Tau protein (p-tau; AFG Scientific, Northbrook, IL, USA); (Catalog # EK720961). Interleukin-1β (IL-1β; BT Lab, Shanghai, China); (Catalog # E0119Ra). All chemicals were of the highest commercial quality.

### 2.3. Preparation of Silk Fibroin (Fb)

The extraction of Fb involved separating the structural protein from Bombyx mori silkworm cocoons, following our previously established protocol [42]. Initially, the adhesive sericin layer covering the cocoons was removed through degumming. This was achieved by boiling the cocoons in a 0.5%*w*/*v* sodium carbonate (Na_2_CO_3_) solution at 100 °C for 1 h, effectively dissolving the sericin while preserving the integrity of the fibroin fibers. The remaining Fb fibers were subsequently rinsed three times with HPLC-grade water to remove residual sericin and inorganic salts, followed by air drying.

To dissolve the Fb fibers, a ternary solvent system consisting of calcium chloride (CaCl_2_), water, calcium nitrate (Ca(NO_3_)_2_), and ethanol (EtOH), in a weight ratio of 30:40:5:20 (*w*/*w*/*w*/*w*), was used. The fibers were immersed in this solvent mixture and heated to 90 °C with continuous stirring for 5 min, facilitating the disruption of intermolecular hydrogen bonds, thereby solubilizing the protein. The resulting Fb solution was then subjected to dialysis against deionized water using a dialysis membrane with a molecular weight cutoff of 10,000 Da over 3–5 days at room temperature to remove residual inorganic salts.

Following dialysis, the Fb solution was centrifuged at 18,000 rpm for 30 min at 4 °C to eliminate any undissolved particles and other impurities. The purified Fb solution was then lyophilized (freeze-dried) and stored at 4 °C until further use, to prevent premature gelation and microbial contamination.

### 2.4. Preparation and Optimization of Empagliflozin-Loaded Chitosan-Fibroin/Poly(lactic acid) Nanofibrous Electrospun Scaffolds (EMPA-Loaded Cs-Fb/PLA NFs)

Fibroin (Fb) and PLA solutions (15%*w*/*v*; for both) were prepared by dissolving these polymers in formic acid and chloroform, respectively. Polymeric blends of both solutions were mixed at different volume ratios (Table 1) and stirred at 400 rpm for 3 h until homogeneity was attained. In the case of Cs-Fb/PLA NFs, Cs was dissolved concomitantly with Fb in formic acid prior to blending with PLA solution. For drug-loaded scaffolds (EMPA-loaded Cs-Fb/PLA NFs), EMPA was dissolved in the polymeric blends at varying concentrations (Table 1). All the polymeric blends were electrospun using an electrospinner (MECC, NANON) equipped with a clip spinneret connected to a 20 cm Teflon tube, which led to a 6 mL syringe fitted with a 22 G nozzle positioned 15 cm from the collector [43]. The nanofibers were collected on a 40 mm wide plate covered with aluminum foil. The applied voltage, solution flow rate, and spinneret speed were tuned to obtain continuous NFs. All electrospun scaffolds were processed under room temperature and humidity conditions of 36%.

### 2.5. Characterization of the Electrospun Nanofibrous Scaffolds

#### 2.5.1. Scanning Electron Microscopy (SEM) Imaging

The surface properties of different blended NFs were visualized using a field emission environmental scanning electron microscope (FE-SEM) (Quattro S, Thermo Scientific, Waltham, MA, USA) with an acceleration voltage of 15 kV. To guarantee accurate investigation, the samples were scanned without any coatings [44,45]. The average diameters of different nanofibers were quantified using ImageJ 153 Java 8 software by analyzing around 100 nanofibers randomly.

#### 2.5.2. Swelling Index (%)

In order to assess the moisture absorption capacity of the nanofibrous dressings, the swelling percentage of the electrospun membranes was evaluated. Dry samples were carefully separated and weighed (*W_d_*), then subsequently immersed one by one in a Petri dish containing 10 mL of PBS at pH 6.8, maintained at a temperature of 37 °C. At specified time intervals, wet samples were carefully lifted, and any excess water was delicately absorbed with a towel. Subsequently, the enlarged membranes were subjected to re-weighing (*W_s_*). The percentage of swelling was determined using the subsequent Equation (1):

(1) $$Swelling(\%)=\frac{W_{s}-W_{d}}{W_{d}}\times 100$$

#### 2.5.3. Contact Angle (Surface Hydrophobicity)

The optimization of the surface properties upon developing nanofibrous scaffolds regarding their application is a crucial step. The hydrophilic and hydrophobic properties of the surface significantly affected the interaction between the synthetic surface and living tissue.

The hydrophobicity of the scaffolds’ surfaces was determined by measuring the corresponding contact angle as previously described [46]. The contact angle (θ) was measured using a face contact angle meter (OCA 20, DataPhysics, Filderstadt, Germany) via the sessile drop technique. An ultra-pure water droplet was dropped onto the scaffold surface using a 0.5 mL precision syringe (Hamilton, Bonaduz, Switzerland). The droplet image was captured with a high-resolution video camera, and its contour was numerically analyzed and fitted to the Laplace-Young equation to determine the contact angle. Experiments were carried out in triplicate for each sample, and the average data were analyzed.

#### 2.5.4. Moisture Loss

Moisture content and water solubility were determined by the methods previously described [46], with some modifications. Briefly, squares of 5 cm length were used to perform these analyses. Samples were weighed before and after being placed in desiccators with dehydrated CaCl_2_ for three days. Moisture content was determined based on the following Equation (2):

(2) $$\mathrm{Moisture}\;\mathrm{content}\;\%\;=\;\frac{Mi-Mf}{Mi}\;\times 100$$

where Mi and Mf are the weights before and after drying, respectively.

#### 2.5.5. Tensile Strength

The tensile strength of various scaffold compositions was assessed using a standard uniaxial tensile test (Z050, Zwick Roell AG, Ulm, Germany). The NFs were sectioned into rectangular dimensions of 40 × 10 mm and placed within paper frames for protection prior to the final tensile testing. Stress–strain curves were obtained by elongating specimens at a rate of 10 mm/min, starting from an initial length of 20 mm under a cell load of 50 N [47]. Measurements were conducted on a minimum of six specimens for each composition to derive average values and standard deviations. The mechanical parameters of scaffolds, including maximum strength and elongation-at-break (%), were calculated as per the following Equations (3) and (4). Three replicates of each sample were examined.

(3) $$TS\left(MPa\right)=\frac{Maximum\;load(N)}{Initial\;cross-sectional\;area\;(m2)}$$

(4) $$EB\left(\%\right)=\frac{Final\;length\;at\;the\;point\;of\;sample\;breakage-Initial\;length\left(mm\right)}{Initial\;length\;of\;the\;sample\;\left(mm\right)}\times 100$$

#### 2.5.6. Nanofibers’ Bulk pH

To directly measure the nanofibrous scaffolds’ pH, a fixed weight of the varying scaffolds was immersed in 5 mL phosphate-buffered saline (PBS) pH 6.8 for two hours. The pH of the solution was then measured using a pH meter (Genway, 3510, Stone, UK). This could give an indication of the bulk pH rather than the surface-specific pH [48].

#### 2.5.7. FTIR

Fourier transform infrared spectroscopy (FT-IR) was analyzed by an FT-IR spectrometer instrument (Bruker Vertex 70, Bremen, Germany) to examine the chemical composition and distinctive fingerprints of the nanofibrous scaffolds throughout the spectrum range of 4000–400 cm^−1^ [49].

#### 2.5.8. XRD

X-ray diffraction (XRD) patterns of the NFs were achieved with an XRD diffractometer (Malvern PANalytical, Malvern, UK) with Cu Kα radiation. The diffraction data were obtained as intensity versus 2θ (5–80°) with a step size of 0.02 and 0.5 s [50].

#### 2.5.9. Drug Content

The concentration of EMPA within the optimized nanofibrous scaffold was assessed through a spectrophotometric technique. 10 mg of the nanofibrous membrane was extracted utilizing 500 µL of the solvent system (formic acid and chloroform) and allowed to stand overnight to guarantee the thorough dissolution of the polymers and the liberation of any encapsulated drug. The aliquot volume of the preceding solution was subjected to dilution with ethanol and subsequently quantified at the peak wavelength of 224 nm, employing a pre-established EMPA calibration curve in absolute ethanol, utilizing UV-vis spectroscopy (Model UV-1601 PC, Shimadzu, Kyoto, Japan). As a result, the calculation for the drug content was conducted using the following Equation (5):

(5) $$Drug\;content=\frac{Amount\;of\;drug}{Weight\;of\;sample}$$

#### 2.5.10. In Vitro Drug Release and Kinetic Modeling

The liberation of EPMA from the optimized nanofibers was conducted for 24 h, involving the suspension of 10 mg of nanofibrous scaffolds in 100 mL of phosphate-buffered saline (PBS) at a pH of 6.8, maintained at a temperature of 37 ± 0.5 °C in a shaking water bath operating at 100 strokes per minute. At designated time intervals of 0.25, 0.5, 1, 2, 4, 6, 8, and 24 h, samples of 2 mL were extracted and subsequently substituted with an equivalent volume of fresh release media. The spectrophotometric determination of released EMPA concentration was conducted utilizing a standard calibration curve, and the cumulative amounts were subsequently plotted against time (t). This experiment was similarly carried out utilizing a comparable quantity of EMPA suspension. The release data were analyzed using various kinetic models to investigate the primary mechanisms governing the drug’s release from the engineered scaffolds.

### 2.6. In Vivo Study

#### 2.6.1. Experimental Design (Table S1)

Rats were randomly divided into six groups (8 rats/group) as follows:

- **Group I:** negative control, received 1 mL/kg saline for 21 days.
- **Group II:** positive control, received AlCl_3_ (100 mg/kg) for 21 days orally [51].
- **Group III:** received 20 mg/kg of pure EMPA orally over 21 days [52].
- **Group IV:** received 5 mg/kg of memantine (standard drug) over 21 days orally [53,54].
- **Group V:** received plain Cs/Fb/PLA-NFs over 21 days (buccal adhesive films).
- **Group VI:** administered 20 mg/kg of EMPA-Cs/Fb/PLA-NFs (buccal adhesive films) over 21 days.

AlCl_3_ was given concurrently with the specific treatments for 21 days to the groups (III-VI). Upon completion of the experiment, animals were used to perform behavioral studies to assess the spontaneous locomotor activity and cognitive ability using an activity cage (Ugo-Basile, Gemonio, Italy) and T-maze. The T-maze used in this study was custom-built from opaque acrylic sheets (black color) to minimize external visual cues. The apparatus consisted of a start arm (30 cm long × 10 cm wide) and two goal arms (each 30 cm long × 10 cm wide), forming a T-shaped structure. All arms had walls 20 cm high to prevent the animals from escaping. The maze was raised 50 cm above the floor utilizing a metal support. At the end of the experiment, the rats were sacrificed under anesthesia and their brains were extracted. One portion of the brain was stored at −80 °C for the measurement of Aβ, p-tau, and IL-1β using ELISA kits. Another portion of the brain was stored at −80 °C for the measurement of amyloid-β (Aβ), phosphorylated tau protein (p-tau), and interleukin-1β (IL-1β) using ELISA kits. A separate portion of the brain tissues was preserved for RT-PCR quantification of Advanced Glycation End-product Receptor (AGER) expression. All parameters were assessed per the manufacturer’s specific guidelines. The last portion of the brain was kept in formalin for histological examination.

#### 2.6.2. Tissue Sample Preparation

##### ELISA Tests

The brain was homogenized using a MPW-120 homogenizer (Med instruments, Warsaw, Poland) in cold PBS and kept overnight at –80 °C. A Sigma and Laborzentrifugen (Osterode am Harz, Germany) 2k15 cooling centrifuge spun the homogenates for 5 min at 5000× *g*. The supernatant was kept at −80 °C for assessment of Aβ, p-tau, and IL-1β using ELISA kits according to specific manufacturer instructions.

##### Real-Time Polymerase Chain Reaction (PCR) Quantification of Advanced Glycation End-Product (AGE) RNA Expression

Total RNA was extracted from cell lysate using Direct-zol RNA Miniprep Plus (Cat# R2072, ZYMO RESEARCH CORP, Irvine, CA, USA). The quantity and quality were subsequently assessed with a Beckman dual spectrophotometer (Tampa, FL, USA). The SuperScript IV One-Step RT-PCR kit (Cat# 12594100, Thermo Fisher Scientific, Waltham, MA, USA) was employed for the reverse transcription of extracted RNA, subsequently followed by PCR in a single step. The primer sequences for the target genes (AGE receptors) and the reference housekeeping gene (GAPDH) are presented in Table 2.

#### 2.6.3. Behavioral Studies

##### Activity Cage

Rodents are individually placed in automated activity cages equipped with infrared beam sensors (or photocell beams) to monitor spontaneous locomotor activity. The system detects horizontal and/or vertical movements (e.g., rearing) by counting the number of beam breaks over a fixed time period. Animals are habituated to the testing room before the experiment to minimize stress-induced activity changes. Activity is recorded and analyzed using compatible software [55,56].

##### T-maze

The T-maze comprises a start arm and two goal arms arranged in a T-shape. The test is typically used to assess spontaneous alternation behavior or spatial working memory. In a spontaneous alternation task, the rodent is placed in the start arm and allowed to choose one of the goal arms freely. After exploration, it is returned to the start position, and a new trial begins. An alternation is recorded when the animal chooses the opposite arm from the previous trial. For rewarded alternation tasks, one goal arm is baited with a reward (e.g., food), and learning or memory is assessed by the animal’s ability to remember and choose the correct arm over repeated trials. The alternation% is calculated using the following Equation (6) [57]:

(6) $$Alternation\%\;=\;\frac{Number\;of\;alternations\;}{Number\;of\;choices-2}\;\times 100$$

#### 2.6.4. Histopathological Studies

Brain tissues were fixed in a 10% neutral buffered formalin solution for 2 days, and then they were dehydrated through ascending grades of ethanol, cleared in xylene, and embedded in paraffin blocks. Serial sections, each 5 μm thick, were mounted on glass slides, subjected to washing in a water bath, and subsequently placed in an oven for dewaxing. The sections were stained using hematoxylin and eosin. Histological changes were evaluated using an electrical light microscope (Olympus CX 41 RF, Tokyo, Japan) and analyzed with Adobe Photoshop version 8.0 in the Department of Pathology, National Research Center, Egypt.

#### 2.6.5. Immunohistochemical Staining

Synaptophysin is a marker for synaptic density and integrity. Using the initial antibody, mouse monoclonal antibody against synaptophysin (CAT #: A0010) was supplied by Lab Vision Laboratories (Fermont, CA, USA). The avidin-biotin peroxidase system was used to evaluate immunohistochemical responses. The surface of the neuronal cell bodies showed brown staining, which suggested positive results for synaptophysin.

#### 2.6.6. Statistical Analysis

In vitro experiments were conducted in triplicate, and the data are presented as mean ± SD. Statistical in vivo analysis was conducted using one-way ANOVA, with subsequent confirmation through Tukey–Kramer’s test. The data are presented as mean ± SE. *p* > 0.05 is considered statistically significant. Statistics were collected using GraphPad Software Prism 8 (San Diego, CA, USA).

## 3. Results

### 3.1. Optimization of Spinning Parameters and Polymeric Composition of Blank and EMPA-Loaded Nanofibrous Scaffolds

#### 3.1.1. Electrospinning Parameters of Pure Fb and Fb/PLA Polymeric Blends

Varying electrospinning parameters were examined to ensure successful NF fabrication using polymeric blend compositions. The optimized electrospinning parameters used for the fabrication of Fb, Fb/PLA, and Cs-Fb/PLA are represented in Table 3. In our study, pure Fb-NFs were successfully electrospun using Fb solution (15%*w*/*v*) in formic acid by applying a relatively high voltage (24 Kv) with a feeding rate of 0.5 mL/h, at a distance of 15 cm from the stationary collector, which was in accordance with the previously reported trials [58,59]. Pure Fb electrospun nanofibers exhibited bead formation and rough surfaces. Thus, to fabricate uniform nanofibrous scaffolds, PLA was added to the electrospun solution in different volume ratios of Fb/PLA: 2:1, 1:1, and 1:2.

Practically, electrospinning of blended PLA and Fb solutions was attained by applying even higher voltages (around 28 kV) and faster feeding rates (0.9 and 0.6 mL/h), greatly enhancing the spinning process’s stability.

#### 3.1.2. Effect of Different Formulation Compositions on the Nanofibers’ Geometry

##### The Impact of the Fb:PLA Volume Ratio

**Figure 1:**
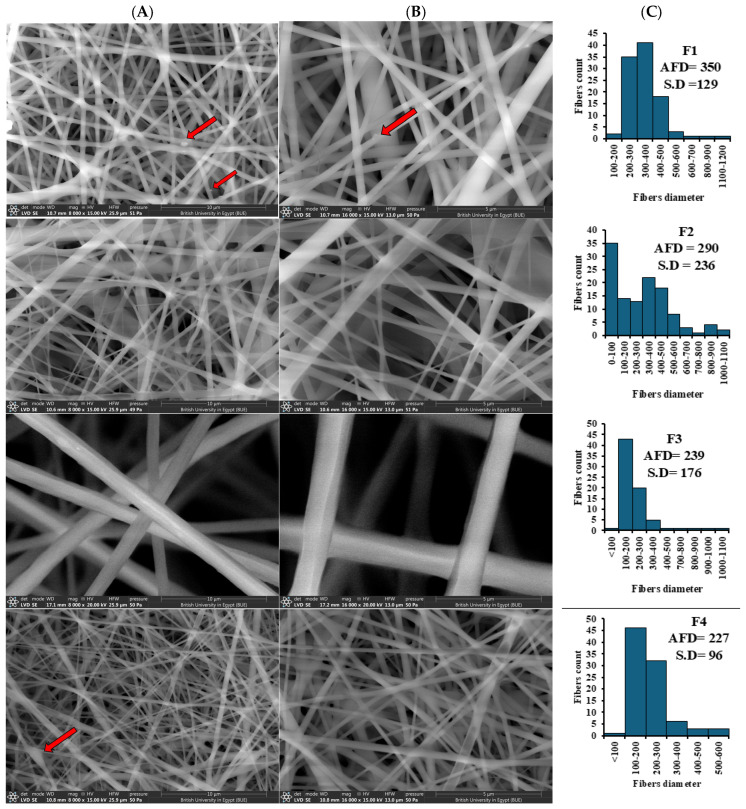
The SEM image expresses the surface morphology of the (F1–F4) nanofibers: (**A**) scale bar = 5 μm, magnification = 8000×; (**B**) scale bar = 10 μm, magnification = 16,000×, and a high voltage (HV) of 15 kV. (**C**) The histograms correspond to the presumed AFDs. Red arrows refer to beads formation.

The impact of the polymeric electrospun solution’s composition using varying volume ratios of both Fb and PLA solutions, namely 1:0, 1:1, 1:2, and 2:1, was studied in terms of the average diameters and size distribution of the fabricated fibers, surface properties and morphology (Figure 1). The SEM micrograph confirmed the formation of pure Fb NFs with some beads apparent and an average fiber diameter (AFD) = 350 ± 129 nm. Blending PLA with Fb significantly impacted the morphology and AFD of the nanofibrous scaffolds (Figure 1). Figure 1C showed the diameter distribution of electrospun nanofibers, and the effect of PLA content on the fiber diameters. Significant reduction (*p* < 0.05) of the AFD was achieved within all the investigated volume ratios (239, 290, and 227 nm) compared to the pure Fb NFs (350 nm), along with diminished bead formation at higher PLA ratios (Fb:PLA; 1:1 and 1:2).

##### The Effect of Cs Incorporation

**Figure 2:**
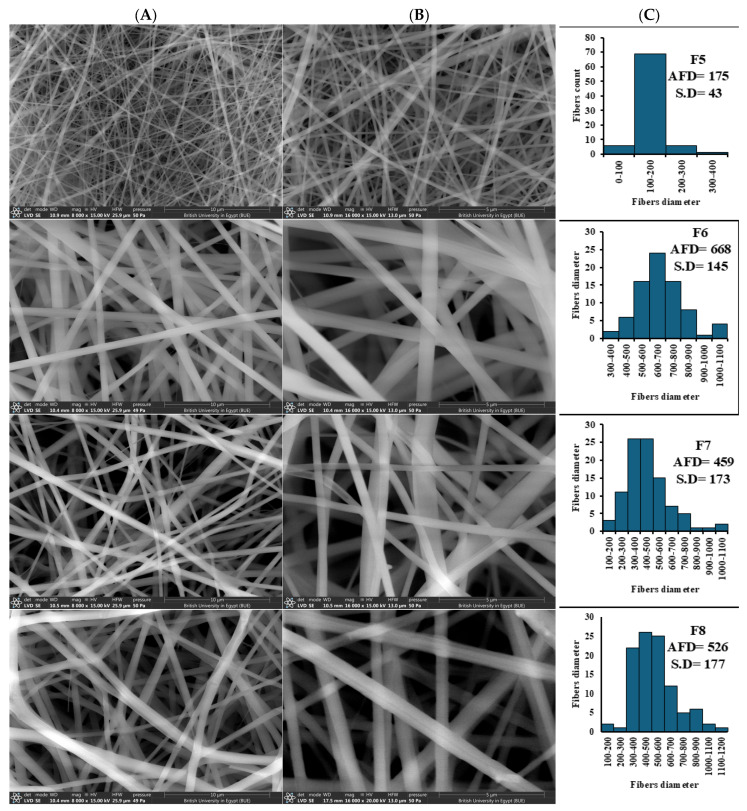
The SEM image expresses the surface morphology of the (F5–F8) nanofibers: (**A**) scale bar = 5 μm, magnification = 8000× and (**B**) scale bar = 10 μm, magnification = 16,000×, and a high voltage (HV) of 15 kV. (**C**) The histograms correspond to the presumed AFDs.

The introduction of Cs at a low concentration (1%*w*/*v*) into the Fb/PLA blend solution (2:1) for electrospinning (F5) significantly altered the geometrical properties of the resulting nanofibers (Figure 2). This is demonstrated by a notable reduction in AFD from 227 nm to 175 nm, accompanied by a more uniform size distribution (Figure 2C).

##### The Effect of Drug Incorporation

The addition of EMPA to the Cs-Fb/PLA blend solution at increasing concentrations of 1%, 3%, and 5%*w*/*v* for electrospinning in the fibrous scaffolds F6, F7, and F8, respectively, was observed for its influence on the fibers’ morphology (Figure 2). The drug incorporation resulted in a notable increase (*p* < 0.05) in the AFD (668.48 ± 174.70, 459.40 ± 172.78, and 526 ± 177 nm, respectively) relative to the corresponding blank nanofibers. In addition, the morphology of the NFs was better, and they displayed a smooth surface area.

#### 3.1.3. Swelling Behavior

**Figure 3:**
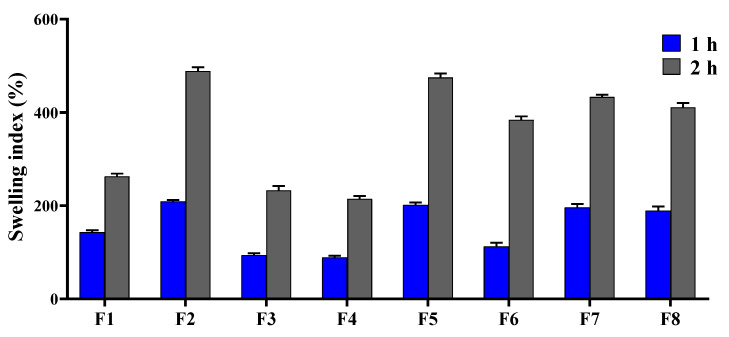
Swelling behavior of various fabricated electrospun NFs.

The swelling index of the Fb/PLA nanofibers (NFs) (F4) was 215 ± 6% after 2 h. Upon incorporation of chitosan (Cs), the swelling index significantly increased to 475 ± 9%, reflecting the influence of Cs addition (Figure 3). Following drug loading at concentrations of 1%, 3%, and 5%, the swelling index of the buccal Cs-Fb/PLA NFs decreased from 475 ± 9% to 384 ± 7%, 434 ± 5%, and 411 ± 9%, respectively.

#### 3.1.4. Contact Angle

**Figure 4:**
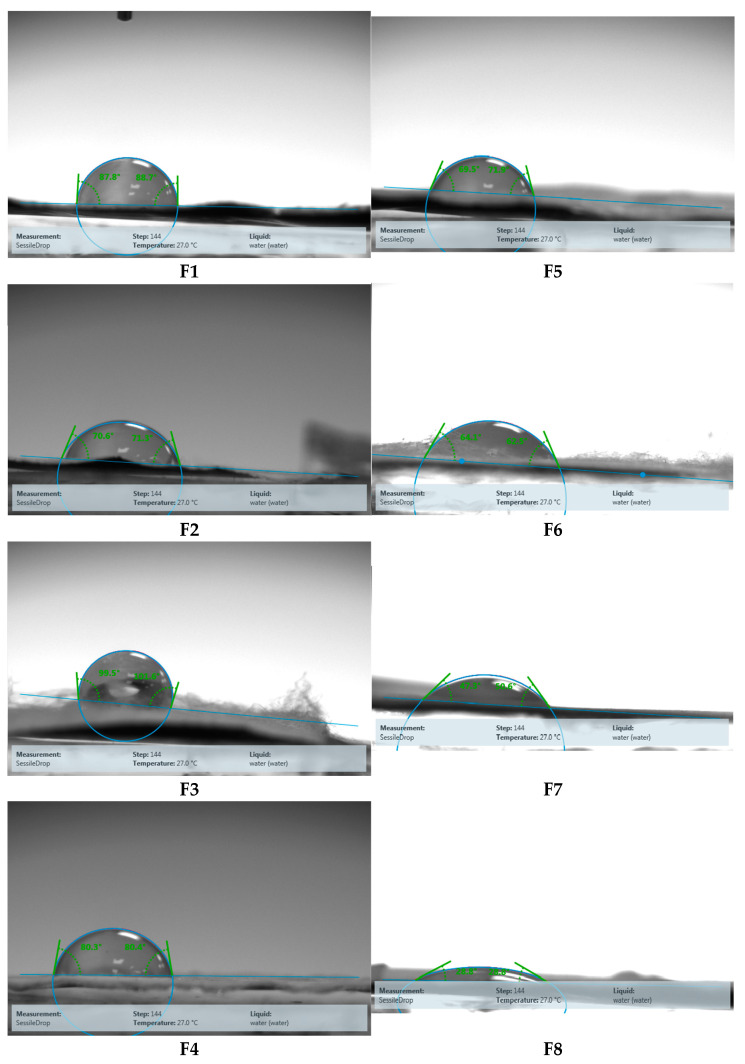
Contact angle measured by the sessile drop method for the different electrospun NFs.

Contact angle measurements were used to evaluate the surface wettability of the electrospun nanofibrous scaffolds. All fabricated scaffolds exhibited hydrophilic behavior (contact angle < 90°), except for sample F3 (Fb:PLA 1:2), which showed a higher water contact angle of 101° (Figure 4).

A non-monotonic trend in contact angle was observed across Fb:PLA ratios. The contact angle decreased from 88° for pure Fb nanofibers to 80° and 70° for Fb:PLA ratios of 2:1 and 1:1, respectively, followed by a sharp increase to 101° at the 1:2 ratio, which reflects the competing surface chemistry and morphological effects driven by compositional shifts.

#### 3.1.5. Moisture Loss

Moisture loss in the Fb-based nanofibers increased with higher PLA ratios and decreased with higher drug concentrations, as shown in Table 4. Nanofibers with greater PLA content exhibited more pronounced moisture loss during drying or thermal analysis. Conversely, samples with higher drug loading showed reduced moisture loss, reflecting the composite materials’ intrinsic physicochemical properties and their interactions with water molecules.

#### 3.1.6. Mechanical Strength

Mechanical testing (Table 4) revealed that pure Fb nanofibers (F1) exhibited tensile strength of 0.83 ± 0.03, while the Fb/PLA NFs at ratios of 2:1, 1:1, and 1:2 displayed tensile strengths of 1.89 ± 0.18, 2.26 ± 0.14, and 2.98 ± 0.17, respectively. However, while tensile strength improved, the elongation-at-break decreased with increasing PLA content, indicating reduced ductility of the nanofibers.

#### 3.1.7. The pH of the Nanofibrous Scaffolds

Bio simulation of the physiological pH range of the buccal mucosa is a critical parameter for ensuring patient comfort and safety. The designed scaffolds exhibited bulk pH values ranging from 6.70 to 7.11. The nanofibers (NFs) were engineered to align with the physiological pH of the buccal mucosa that could mitigate the risk of mucosal irritation or tissue damage associated with substantial pH deviations.

#### 3.1.8. FTIR

**Figure 5:**
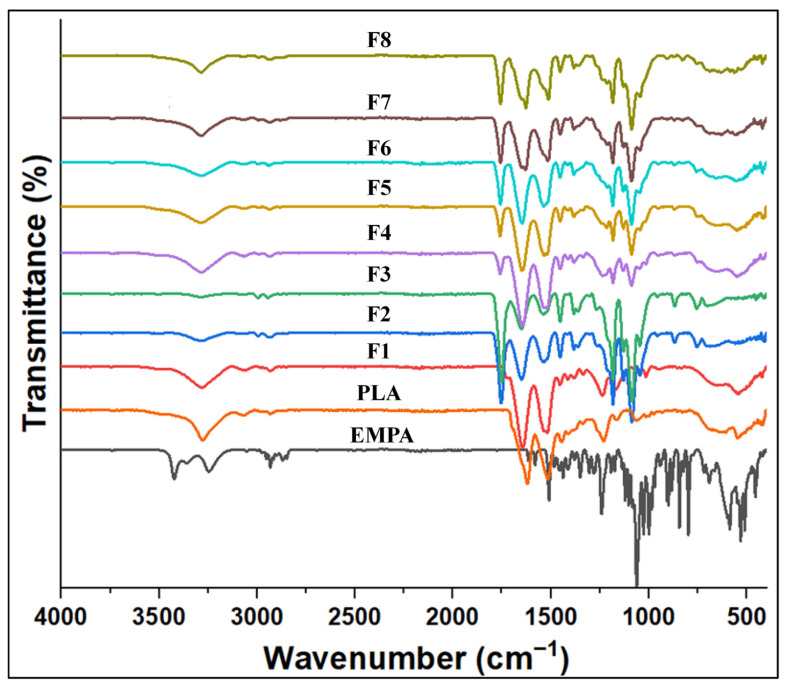
FTIR spectra showing chemical composition for EMPA, PLA, F1, F2, F3, F4, F5, F6, F7, and F8.

FT-IR analyses were conducted to investigate the physical and chemical interactions among the components of the synthesized nanofibers (NFs) [45]. Figure 5 presents the FTIR spectra of the various NF scaffolds and their respective components, highlighting distinctive absorption peaks that reflect the functional groups present in EMPA, Fb, and PLA. The FTIR spectrum of EMPA reveals several significant peaks that correspond to its functional groups, with the peak at 3400 cm^−1^ indicating O–H stretching, suggesting the presence of hydroxyl groups that can form hydrogen bonds and enhance the material’s stability. The peaks at 3253 cm^−1^ and 3050 cm^−1^ are associated with aromatic C–H stretching, signifying the presence of aromatic structures. The peak at 2900 cm^−1^ corresponds to aliphatic C–H stretching, reflecting the presence of alkyl chains that influence the scaffold’s flexibility and hydrophobic characteristics, while the peak at 998 cm^−1^ is attributed to C–O stretching, which is crucial for understanding the chemical interactions within the polymer matrix [60]. The FTIR spectrum of Fb showcases characteristic bands primarily associated with peptide bonds, with the Amide I Band at 1650 cm^−1^ related to C=O stretching vibrations from the backbone amide bonds, providing insight into the protein’s secondary structure. The Amide II Band at 1530 cm^−1^ corresponds to N–H bending and C–N stretching, confirming the presence of peptide linkages, while the Amide III Band at 1300 cm^−1^ arises from the in-phase combination of C–N stretching and N–H in-plane bending, reflecting the protein’s structural characteristics. A broad absorption band at 3300 cm^−1^ is assigned to N–H (amide A) and O–H stretching vibrations, indicating hydrogen bonding, which is vital for maintaining the structural integrity and stability of the protein. Additionally, peaks at 2900 cm^−1^ and 2950 cm^−1^ represent C–H asymmetric stretching from methyl groups and the methylene backbone, respectively [61].

Regarding the index peaks of PLA, the presence of strong absorption peaks at about 1700 cm^−1^ and 1200 cm^−1^ for PLA is due to the C=O tensile vibration and C–O stretching bands [62]. All blended scaffolds with varying ratios exhibited new combination peaks at 1700 cm^−1^, and those containing EMPA displayed specific vibrational peaks at 900 cm^−1^, 1300 cm^−1^, and 1650 cm^−1^.

#### 3.1.9. Crystallography Investigation for Unloaded and Loaded Cs/Fb/PLA Nanofibrous Scaffolds (XRD)

**Figure 6:**
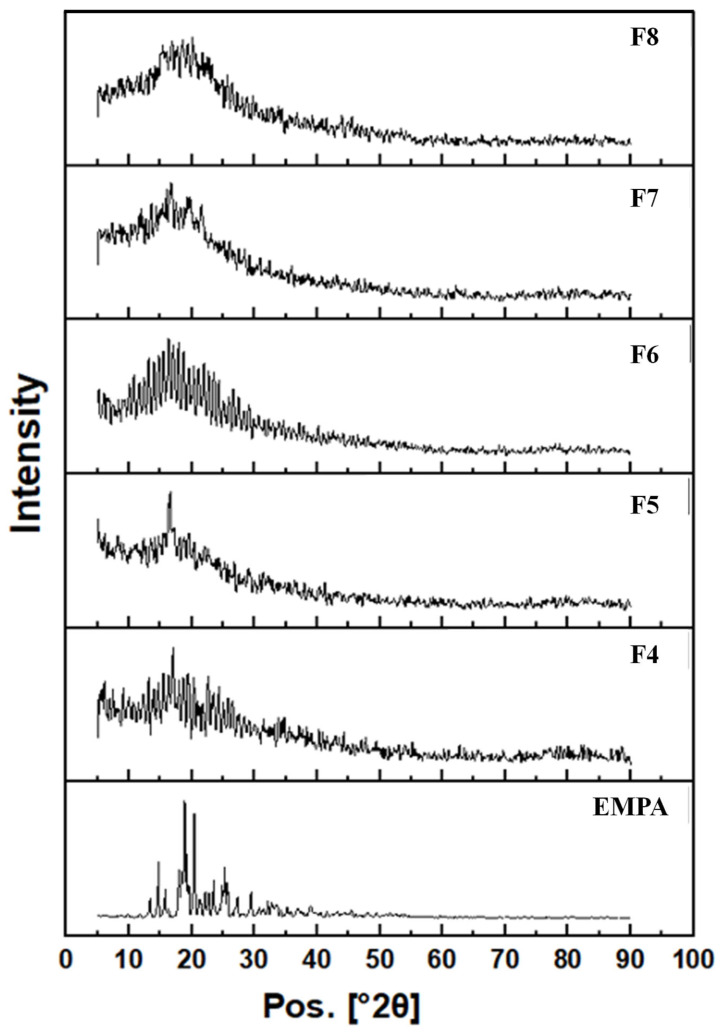
XRD analysis for EMPA, F4, F5, F6, F7, and F8.

X-ray diffraction (XRD) patterns were employed to investigate the crystallographic structure of various nanofibrous scaffolds, including EMPA, F4, F5, F6, F7, and F8 NFs. The EMPA powder exhibited a range of distinct intensity bands at diffraction angles (2θ) of 15°, 17°, 21°, 23°, 26°, and 30°, indicating its crystalline structure, as illustrated in Figure 6 [63]. As a result of the electrospinning process, all fabricated nanofibrous scaffolds displayed a significantly reduced level of crystallinity, with the absence of EMPA’s characteristic diffraction peaks, even at the highest EMPA concentration of 5%.

#### 3.1.10. Drug Content

All the drug-loaded Cs/Fb/PLA NFs exhibited remarkably high drug content values (Table 4). As the concentration of the drug incorporated into the polymeric electrospun solution increased, the drug content value of the corresponding NF scaffolds increased.

#### 3.1.11. In Vitro Release Kinetics

**Figure 7:**
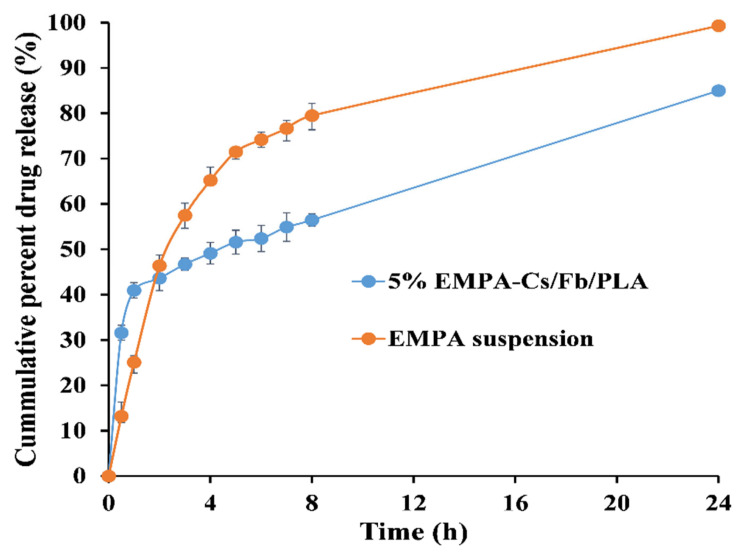
In vitro release profiles for optimized EMPA-Cs/Fb/PLA NFs and EMPA suspension.

The EMPA release from Cs/Fb/PLA NFs displayed a biphasic pattern, as shown in Figure 7. After 30 min., about 30% of the EMPA loaded into the NFs was liberated. Afterwards, a more sustained release pattern was attained, where the cumulative amount of EMPA released reached 56% and 85% after 8 h and 24 h, respectively.

Kinetic model fitting (Figure S1) showed the highest correlation with the Korsmeyer–Peppas model (R^2^ = 0.97, n = 0.255) and the Weibull model (R^2^ = 0.95, β = 0.0363). The Higuchi model displayed a lower correlation (R^2^ = 0.885), although it was still indicative of diffusion-controlled behavior.

### 3.2. In Vivo Study

#### 3.2.1. Assessment of Aβ–AGER–p-tau Axis and Pro-Inflammatory Cytokines (IL-1β)

**Figure 8:**
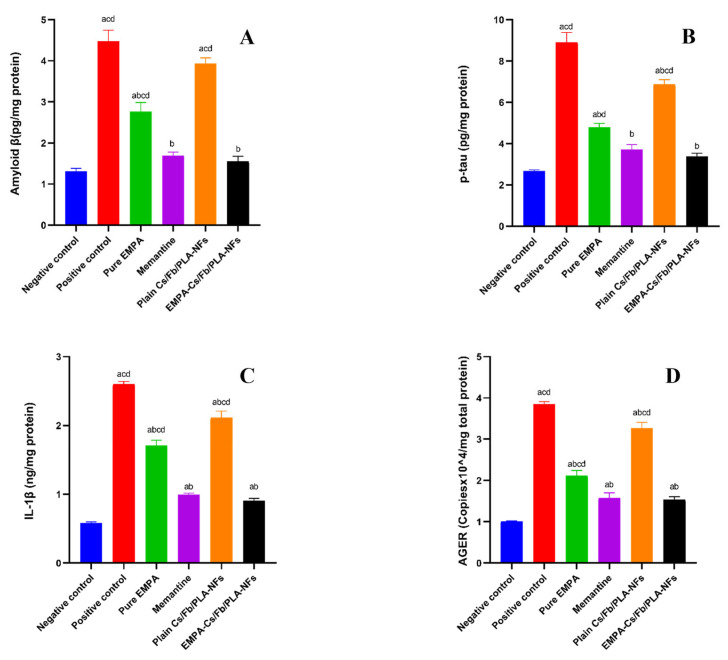
Effect of pure EMPA and EMPA-Cs/Fb/PLA-NFs (20 mg/kg; p.o for 21 days) on amyloid-β (**A**), p-tau (**B**), IL-1β concentration (**C**) and AGE receptors expression in brain tissue (**D**), compared to memantine (5 mg/kg), AlCl_3_ induced Alzehiemer’s disease (AD) in Wistar rats. All data are presented as mean ± SE. (n = 8). a: *p* < 0.05 significant from negative control group. b: *p* < 0.05 significant from positive control group. c: *p* < 0.05 significant from memantine (standard) group. d: *p* < 0.05 significant from EMPA-Cs/Fb/PLA-NFs.

Our study revealed that AlCl_3_ (100 mg/kg) administration for 21 days to the positive control group led to a significant (*p* < 0.05) increase in Aβ and p-tau concentrations, with 3.45- and 3.34-fold increases, respectively, compared to the negative control group. The oral administration of AlCl_3_ significantly (*p* < 0.05) elevated the RNA expression of AGE receptors in brain tissues, exhibiting a 3.8-fold increase compared to the negative control group. Moreover, our results demonstrated a significant (*p* < 0.05) elevation in IL-1β, with a 4.48-fold increase after administration of AlCl_3_ compared to the negative control group. The present study also demonstrated that oral administration of memantine (5 mg/kg) for 21 days alongside AlCl_3_ significantly reduced Aβ, p-tau, and IL-1β, and also attenuated AGE receptor expression, with significant (*p* < 0.05) 2.66-, 2.41-, 2.62-, and 2.43-fold decreases as compared to the positive control group. We have also declared that the administration of EMPA-Cs/Fb/PLA-NFs (buccal adhesive films) (20 mg/kg) for 21 days concurrently with AlCl_3_ for 21 days has exerted a significant protective role against AD, which was demonstrated by the significant amelioration of Aβ, p-tau, and IL-1β concentration, as well as the attenuation of AGE receptor expression in brain tissue with significant (*p* < 0.05) 2.88-, 2.64-, 2.87-, and 2.5-fold decreases as compared to the positive control group, and significant (*p* < 0.05) 2.54-, 2.04-, 2.41-, and 2.13-fold decreases as compared to the plain Cs/Fb/PLA-NFs group. EMPA-Cs/Fb/PLA-NFs resulted in significant reductions in Aβ, p-tau, and IL-1β concentrations, as well as a decrease in AGE receptor expression in brain tissue. Specifically, the reductions were 1.78-, 1.42-, 1.89-, and 1.38-fold, respectively, when compared to the group receiving pure EMPA, with statistical significance (*p* < 0.05). The results recorded no significant difference (*p* < 0.05) between EMPA-Cs/Fb/PLA-NFs and the standard drug, memantine, utilized in the treatment protocol for AD. Data are depicted in Figure 8.

#### 3.2.2. Behavioral Studies

##### Activity Cage

**Figure 9:**
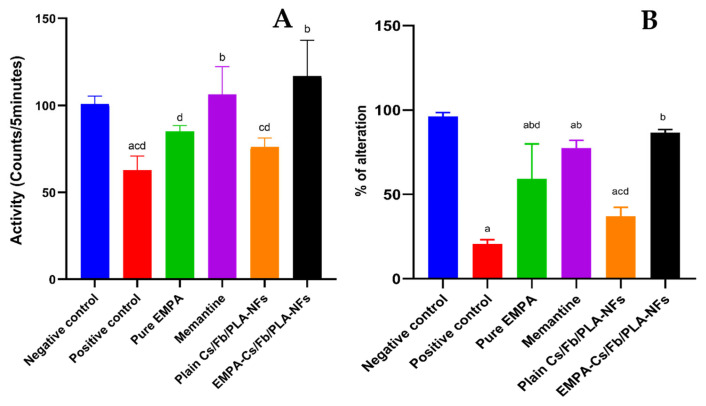
(**A**) Effect of pure EMPA and EMPA-Cs/Fb/PLA-NFs (20 mg/kg; p.o for 21 days) on spontaneous locomotor activity and compared to memantine (5 mg/kg). AlCl_3_ induced AD in Wistar rats. All data are presented as mean ± SE. (n = 8). (**B**) Effect of pure EMPA and EMPA-Cs/Fb/PLA-NFs (20 mg/kg; p.o for 21 days) on continuous spontaneous alteration behavior was assessed utilizing the T-maze apparatus and compared to memantine (5 mg/kg). AlCl_3_ induced AD in Wistar rats. All data are presented as mean ± SE. (n = 8). a: *p* < 0.05 significant from negative control group. b: *p* < 0.05 significant from positive control group. c: *p* < 0.05 significant from memantine (standard) group. d: *p* < 0.05 significant from EMPA-Cs/Fb/PLA-NFs.

The present investigation demonstrated that oral administration of AlCl_3_ (100 mg/kg) for 21 days to the positive control group led to a substantial (*p* < 0.05) decrease in spontaneous locomotor activity of the rats, with a 1.61-fold decrease, compared to the negative control group. The results also indicated no significant difference (*p* < 0.05) between plain Cs/Fb/PLA-NFs and the positive control group. Administration of memantine (5 mg/kg) concurrently with AlCl_3_ for 21 days led to a significant (*p* < 0.05) increase in spontaneous locomotor activity, with a 1.69-fold increase as compared to the positive control group. Administration of EMPA-Cs/Fb/PLA-NFs (20 mg/kg) for 21 days concurrently with AlCl_3_ showed a significant increase in spontaneous locomotor activity in rats as compared to the positive control group as well as the group administered with pure EMPA in a 1.86- and 1.38-fold increase, respectively. No significant difference was reported between group administered EMPA-Cs/Fb/PLA-NFs and group administered the standard drug (memantine). Data are depicted in Figure 9A.

##### T-maze Test

Our study revealed that oral administration of AlCl_3_ (100 mg/kg) over a period of 21 days led to a significant (*p* > 0.5) decline in cognitive ability, evidenced by a 4.64-fold reduction in the alternation behavior of rats in the T-maze test relative to the negative control group. The findings revealed no significant difference (*p* < 0.05) between plain Cs/Fb/PLA-NFs and the positive control group. Oral treatment of rats with memantine (5 mg/kg) for 21 days concurrently with AlCl_3_ for 4 successive weeks significantly (*p* > 0.5) improved the cognitive deterioration induced by AlCl_3_ by increasing the alteration % by a 3.74-fold increase as compared to the positive control group. Administration of EMPA-Cs/Fb/PLA-NFs (20 mg/kg) for 21 days concurrently with AlCl_3_ showed a significant increase of alteration % in rats as compared to the positive control group, as well as the group administered with pure EMPA, with a 4.17- and 1.46-fold increase, respectively. No significant difference was reported between the group administered EMPA-Cs/Fb/PLA-NFs and the group administered the standard drug (memantine). Data are depicted in Figure 9B.

#### 3.2.3. Histopathological Studies

**Figure 10:**
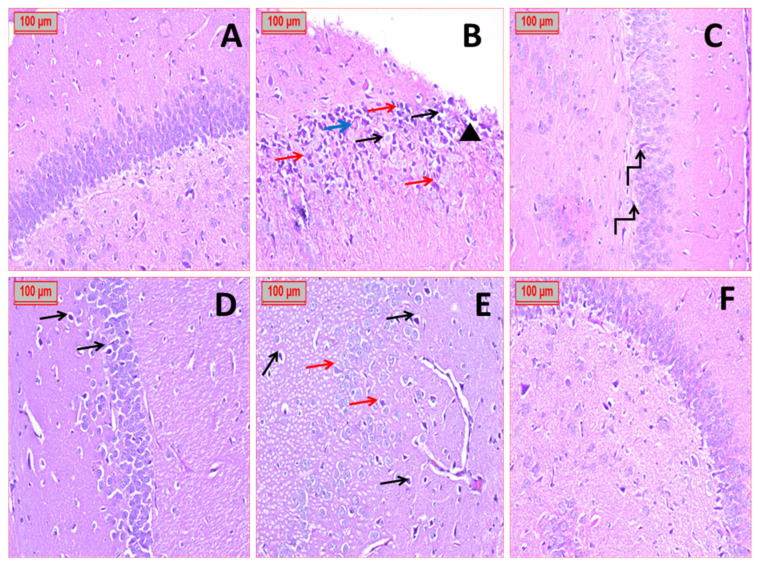
Photomicrographs of H&E-stained hippocampus area from (**A**) the negative control showing normal neurons arranged in a band; (**B**) the positive control (AlCl_3_) group; (**C**) the pure EMPA group; (**D**) the standard (memantine) treated group; (**E**) the plain Cs/Fb/PLA-NFs group; and (**F**) the EMPA-Cs/Fb/PLA-NFs group. Black arrow: neuro-degenerated pyramidal cells with pyknotic nuclei and surrounded by pericellular space. Red arrow: shrunken necrotic neuron with pyknotic nuclei. Blue arrow: amyloid. Broken arrow: neuro-degenerated cells appear as flame-like with pointed ends (H&E, ×200).

H&E-stained sections were employed for examination of the histological structure of Para sagittal sections of the hippocampus of the negative control adult group, which revealed a part of the hippocampus that was formed of three layers: molecular, pyramidal, and polymorphic. The pyramidal nerve cell layer was thick and composed of closely packed pyramidal cells with vesicular nuclei, prominent nucleoli, and pale basophilic cytoplasm. Scattered neuroglial cells with darkly stained nuclei and normal pericellular halos are present (Figure 10). The positive control group (AlCl_3_-treated) had induced Alzheimer’s with characteristic histopathological changes, as seen in the pyramidal layer, which contained disordered, loosely packed large cells with shrunken, darkly stained nuclei and vacuolated cytoplasm. There were multiple large glial cells with darkly stained nuclei, surrounded by wide, lightly stained spaces. Focal amyloid area and destructed areas were seen. Few pyramidal cells appeared more or less normal, with basophilic cytoplasm and large central rounded vesicular nuclei (Figure 10B). Oral administration of pure EMPA concurrently with AlCl_3_ revealed moderate improvement with the presence of some neurodegeneration (Figure 10C). Rats that were treated with memantine concurrently with AlCl_3_ revealed marked improvement present as a normal hippocampus area with minimal neurodegeneration that appears as pyknotic nuclei and surrounded by pericellular space (Figure 10D). Treatment with plain Cs/Fb/PLA-NFs showed no improvement in the AlCl_3_-treated group, which exhibited disordered, loosely packed pyramidal neuronal cells, mostly with neurodegeneration, with pyknotic nuclei and surrounded by pericellular space (Figure 10E). Rats subjected to AlCl_3_ and treated with EMPA-Cs/Fb/PLA-NFs showed marked improvement that was close to normal (Figure 10F).

#### 3.2.4. Immunohistochemical Staining

**Figure 11:**
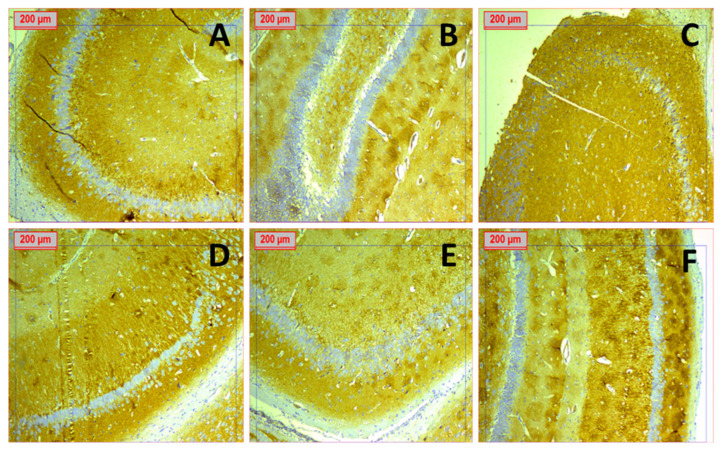
Photomicrograph of synaptic density in the hippocampus identified by synaptophysin (SYN) immunohistochemistry. (**A**) The negative control group showed a strong positive reaction to SYN. (**B**) The positive control group revealed a marked decrease in the immunoreactivity of SYN in the form of faint brown coloration. (**C**) The pure EMPA-treated group showed a marked increase in SYN immunoreactivity. (**D**) The standard (memantine) group reveals a mild increase in SYN immunoreactivity. (**E**) The plain Cs/Fb/PLA-NFs treated group showing faint positivity of SYN. (**F**) EMPA-Cs/Fb/PLA-NFs shows a strong positive reaction of SYN. Synaptophysin, ×100.

Synaptic density was assessed in the hippocampal subregions by synaptophysin immunostaining to identify synaptic vesicles. The expression level of synaptophysin protein can be evaluated by the variation in brown color intensity in micrographs stained using the immunohistochemistry (IHC) approach (Figure 11). Micrographs of the positive control group and plain Cs/Fb/PLA-NFs exhibited diminished synaptophysin expression, shown by a weak brown stain intensity (borderline to weak). In the negative control group, pure EMPA, memantine, and EMPA-Cs/Fb/PLA-NFs sections exhibited pronounced staining intensity, ranging from moderate to strong.

## 4. Discussion

Like numerous natural biopolymers, Fb presents challenges when it comes to electrospinning, and is hence blended with synthetic polymers for a more stable electrospinning process [64]. The backbone of Fb conveys a negative charge, so when subjected to an electrical field, the presence of similarly charged chains leads to repulsion, thereby hindering the efficacy of the electrospinning process and necessitating relatively high voltage application [65].

The integration of PLA with Fb solutions fosters notable molecular interactions that affect the parameters of the electrospinning process and the properties of the resulting fibers. The existence of polar functional groups in Fb, including amide and hydroxyl groups, promotes hydrogen bonding with the ester groups of PLA. These interactions lead to heightened viscosity and increased surface tension. This necessitates the use of elevated voltages in the electrospinning process to counteract the increased surface tension and ensure the stability of jet formation [66]. Furthermore, the Fb/PLA blend solution demonstrates reduced conductivity compared to pure Fb solution, attributed to the inclusion of non-conductive hydrophobic PLA chains that diminish the availability of free charge carriers in the solution [58]. This impacts the charge density on the polymer jet, thereby influencing the dynamics of electrospinning [64,67,68]. Thus, adjustments to electrospinning parameters, including feed rate, are essential, since improper flow rates may result in defects such as bead formation or fiber breakage, thereby jeopardizing fiber uniformity [69]. PLA addition introduces pseudoplastic behavior, necessitating higher shear rates (achieved via increased feeding rates) to maintain consistent polymer flow through the syringe needle. This adjustment compensates for the amplified viscoelastic forces that resist electrostatic stretching [70,71].

Fb demonstrates inherent electrospinnability due to its amphiphilic nature, β-sheet-forming propensity, and tunable molecular weight, which facilitate chain entanglement and solvent-dependent conformational transitions [72]. These characteristics enable Fb to form nanofibers under optimized electrospinning conditions, as evidenced by the SEM-confirmed fibrous structures. However, the observed bead formation in pure Fb NFs typically arises from reduced solution viscosity and impaired jet stabilization [73]. Consequently, the addition of PLA to the Fb solution is attempted to optimize nanofiber morphology and reduce bead formation through synergistic rheological and interfacial interactions [74].

The addition of PLA led to smaller and more uniform fibers, especially at the 2:1 ratio, likely attributed to the semi-crystalline characteristics and hydrophobic properties of PLA that facilitate more regulated rates of solvent evaporation, thereby enhancing stable jet elongation and minimizing the chances of bead formation, which frequently results in thicker and irregular fibers [61,75]. On incorporation of PLA at the lowest volume ratio 2:1 (F4), electrospun NFs exhibited the smallest AFD, and their distribution was the narrowest (227 ± 96 nm). The further increase in the PLA ratio in F2 (Fb:PLA of 1:1) and F3 (Fb:PLA of 1:2) yielded larger AFD (290 and 239 nm, respectively), and broader diameter distributions were noticed. These findings came in great accordance with a previous study, where increasing PLA content above 15% led to thicker fibers and broader diameter distribution [76]. As the PLA ratio in the blend increased to more than 2:1 (Fb:PLA), a distinct polarization in fiber diameters was evident, and the electrospun fiber mats in these instances exhibited friability, indicating a clear phase separation within the electrospun scaffolds [76].

The introduction of Cs led to a reduction in fiber diameter and improved uniformity, attributed to the unique physicochemical characteristics of Cs, which functions as a polyelectrolyte, thereby improving the conductivity and charge density of the solution throughout the electrospinning process. The enhancement of solution conductivity leads to an augmented elongation of the polymer jet when subjected to the electric field, thereby encouraging the creation of finer fibers characterized by more consistent diameters [77]. Nevertheless, this fining of fiber morphology is ascribed to the electrostatic interactions and hydrogen bonding between Cs and Fb molecules [78]. This result agrees with that reported formerly, where Pure Fb NFs had a larger AFD (484 ± 410 nm) than CS/Fb NFs [77]. Additionally, NFs’ diameters decreased with the increasing Cs content [77]. Moreover, incorporating Cs appears to bolster the intermolecular interactions within the Fb-PLA matrix, thereby augmenting chain the entanglement and viscoelasticity elements essential for achieving stable jet formation and ensuring fiber uniformity [79].

Addition of EMPA enhanced the morphology of the NFs. This came in accordance with findings that reported that after adding EMPA to the pure solution, there was a noticeable increase in the diameter (from 311 ± 37 to 674 ± 108 nm) of the PLA/PCL(Poly(ε-caprolactone)) mats [80]. This effect is attributed to drug-polymer interactions that modify solution rheology and jet dynamics. EMPA, a small-molecule drug (Mw: 450.9 g/mol), likely increased solution viscosity through the formation of hydrogen bonds with the amine groups of Cs and hydroxyl/carboxyl moieties in Fb and PLA [80,81]. This interaction enhanced polymer chain entanglement and reduced shear-thinning behavior during electrospinning. The increase in viscosity hindered jet elongation in the electric field, promoting the formation of thicker fibers. This phenomenon was noted in polycaprolactone (PCL) systems, where the incorporation of hydrophobic drugs such as curcumin led to a 35–40% increase in AFD due to diminished polymer chain mobility [81,82]. Moreover, EMPA may interfere with intermolecular interactions between Cs and Fb/PLA, potentially leading to a decrease in solution conductivity and a reduction in electrostatic stretching forces. This mechanism aligns with observations in Cs/polyethylene oxide (PEO) blends containing metformin, where a 15–20% decrease in conductivity was associated with a 25% increase in fiber diameters [83].

Assessing the swelling index of nanofibers employed in buccal drug delivery is essential, as it significantly impacts the nanofiber’s ability to absorb fluids, thereby influencing drug release kinetics, mucoadhesion, and the overall effectiveness of the therapy. The swelling behavior promotes improved adhesion to the moist buccal mucosa, which in turn facilitates an extended residence time and regulated drug diffusion, ultimately enhancing bioavailability and patient compliance [84]. Furthermore, the capacity for swelling plays a crucial role in governing the dissolution and diffusion of drugs within the nanofiber matrix, a factor that is vital for attaining a delayed and sustained release of medication in the oral cavity environment [85]. Thus, evaluating the swelling ratio is a key parameter in optimizing nanofiber formulations for buccal delivery applications. The observed increase in the swelling index of Fb/PLA NFs (214.9% ± 6.1) upon Cs addition (475.2% ± 8.6) is primarily attributed to Cs’s inherent hydrophilicity. Unlike the relatively hydrophobic polylactic acid (PLA) and moderately hydrophilic fibroin, Cs possesses abundant hydrophilic functional groups (hydroxyl and protonated amino groups) that exhibit strong affinity for water molecules, enhancing water uptake into the nanofiber matrix [38].

The subsequent reduction in swelling index after drug loading is likely due to the hydrophobic properties of the EMPA, which naturally lessens the overall hydrophilicity of the nanofiber matrix, consequently leading to a decrease in water absorption [86]. This controlled reduction in swelling can be advantageous for buccal delivery, potentially moderating initial burst release and improving the system’s structural integrity during application.

Contact angle is pivotal in enhancing nanofibrous buccal drug delivery membranes, influencing their wetting characteristics and interfacial interactions. This metric directly impacts saliva’s compatibility, the adhesion to mucosal surfaces, and the drug release kinetics. The meticulous regulation of hydrophilicity and hydrophobicity, as indicated by contact angle measurements, guarantees ideal bioadhesion to the oral mucosa, all while preserving the structural integrity essential for the controlled release of therapeutics [87]. The initial decrease in contact angle with increasing PLA ratio in Fb:PLA nanofibrous scaffolds indicates enhanced surface hydrophilicity. Though PLA is inherently hydrophobic, its integration into a hydrophilic Fb-based matrix creates a composite surface where PLA’s semi-crystalline domains disrupt Fb’s β-sheet packing, exposing polar amino acid residues (-OH, -COOH) at the fiber-air interface [72]. The present findings correspond with the mechanism that PLA alters the hydrophobic crystalline domains of Fb and introduces nanoscale topographical features, collectively surpassing the inherent hydrophobic properties of PLA. This intriguing paradox, in which a hydrophobic additive contributes to an increase in the hydrophilicity of a composite, arises from the prevailing influence of protein-polymer interfacial reorganization, overshadowing the bulk properties of the polymer [88,89]. This phenomenon has also been observed in albumin-PLGA systems [90].

The gradual decrease in contact angle upon increase in PLA content in the corresponding scaffolds could also be attributed to the concomitant reduction in AFD, which in turn reduces porosity of the surfaces and the entrapped air. This could have led to an attenuated Cassie-Baxter wetting state where water droplets sit atop trapped air pockets rather than fully wetting the surface. This reduces the solid–liquid contact area, increasing the apparent contact angle [91]. A similar trend was observed by Dou et al. [92] upon fabrication of PLA fibers and correlated to the reduced diameters of the electrospun fibers. Similarly, collagen-PLA fibers displayed a higher contact angle than pure PLA ones and correlated to the interaction of collagen fibrils with the PLA NFs [93].

As the PLA content in Fb/PLA blend nanofibers increases beyond a critical threshold, typically around 30–50%, a sharp increase in contact angle is observed. This phenomenon is primarily attributed to the dominance of PLA’s hydrophobic methyl groups at the fiber surface, which significantly raises the surface hydrophobicity [94,95]. At higher PLA concentrations, morphological transitions occur, including phase separation, where the efficiency of Fb dispersion decreases and PLA-rich domains with inherently low surface energy become more prevalent [96]. Additionally, increased PLA content leads to greater crystallinity and chain entanglements, which further restrict the exposure of polar groups on the fiber surface, thereby enhancing hydrophobicity [88]. The sharp increase in contact angle is also influenced by the percolation threshold, where PLA forms continuous phases that effectively bury the hydrophilic fibroin, and by weakened interfacial adhesion between Fb and PLA at high PLA content, which exacerbates the overall surface hydrophobicity [97,98,99].

Moisture loss assesses the NF’s capacity to retain moisture and signifies its behavior in arid environments. The increase in moisture loss with rising PLA ratios can be attributed to PLA’s hydrophobic and semi-crystalline nature, which inherently limits water absorption; paradoxically, its increased content in Fb nanofibers can lead to greater moisture loss during drying or thermal analysis due to reduced water retention capacity of the entangled matrix. This phenomenon is consistent with studies reporting that increasing PLA content in protein-based nanofibers reduces overall hydrophilicity, facilitating faster evaporation of surface-bound moisture and resulting in higher measured moisture loss [95]. In contrast, higher drug loading within the nanofibers tends to reduce moisture loss, likely because many drugs, especially hygroscopic compounds, can interact with water molecules, retaining moisture within the fiber matrix through hydrogen bonding or other molecular interactions. Similar observations were reported by Li et al. [100], who found that increased drug concentration in electrospun nanofibers enhanced water retention and decreased moisture loss, attributed to the drug’s affinity for water and its role in stabilizing the fiber microenvironment. Furthermore, the interplay between polymer hydrophobicity and drug hydrophilicity governs the overall moisture dynamics in composite nanofibers, influencing their stability, mechanical properties, and drug release behavior [101]. These findings emphasize the importance of optimizing polymer-to-drug ratios in Fb/PLA nanofibers to tailor moisture-related properties for specific biomedical applications such as wound dressings or controlled drug delivery systems.

Mechanical characterization, especially tensile strength evaluation, is essential for electrospun polymeric blend nanofibers, as it directly influences their functional performance and structural integrity in applications like tissue engineering scaffolds, wound dressings, and drug delivery systems [72]. The increased tensile strength of the fabricated nanofibers with a higher PLA ratio in Fb/PLA blend nanofibers represents a significant and frequently documented phenomenon within biomaterials science, attributable to the interplay of several key material properties and structural factors.

Primarily, this enhancement stems from the superior inherent mechanical strength and stiffness of PLA compared to the relatively more ductile, yet often weaker, regenerated silk fibroin derived from sources like Bombyx mori. PLA is a semi-crystalline synthetic polymer known for its high tensile modulus and strength, whereas regenerated Fb, while biocompatible and flexible, typically exhibits lower tensile strength in its pure electrospun form due to factors like residual solvent, imperfect chain alignment, or the predominance of amorphous structures unless specifically treated [102]. Consequently, as the PLA fraction increases within the blend, it progressively dominates the mechanical response of the composite nanofiber mat, imparting its higher strength characteristics to the overall structure.

Beyond the simple rule of mixtures, research indicates that the increasing PLA ratio facilitates critical microstructural changes contributing to strength enhancement [27]. Blending Fb with synthetic polymers could influence crystallization behavior, and similar interactions are reported with PLA [103]. Increased crystallinity directly correlates with improved tensile strength, as crystalline domains act as physical crosslinks, restricting chain mobility and enhancing load-bearing capacity under tensile stress [27]. Furthermore, at higher PLA ratios, a phase inversion likely occurs, transitioning from a Fb-continuous phase with PLA dispersed domains to a PLA continuous phase with Fb dispersed domains. Once PLA forms the continuous, percolating network throughout the nanofiber mat (typically observed beyond a critical blending ratio, often around 50% or higher, depending on processing), it effectively bears the majority of the applied load [71]. It was specifically noted in Fb/PLA blends that the mechanical properties shifted significantly when PLA became the matrix phase, leading to a substantial rise in tensile strength and modulus [104].

Similar findings, reinforcing the role of a stiffer polymer component in enhancing the tensile strength of natural/synthetic blend nanofibers, are prevalent in the literature. For instance, in Cs/PLA blends, increasing PLA content consistently led to higher tensile strength and modulus due to PLA’s superior mechanical properties and phase continuity [105]. Likewise, studies on gelatin or collagen electrospun mats report analogous trends where the synthetic polymer (PLA and PCL, respectively) imparts mechanical robustness as its ratio increases [28,106]. The phenomenon observed in Fb/PLA blends thus aligns with broader principles governing the mechanical behavior of biopolymer/synthetic polymer composites, where the incorporation of a stronger, stiffer synthetic component like PLA effectively reinforces the natural polymer matrix, leading to measurable improvements in tensile strength, particularly when the synthetic component forms the continuous phase. In contrast to tensile strength, the elongation-at-break was found to decrease significantly upon incorporation of PLA in Fb-based nanofibers.

The reduction in ductility is mainly due to the material properties of PLA and the morphological changes in the blend. PLA has a higher glass transition temperature (Tg) and increased chain stiffness than regenerated Fb, which typically shows a more flexible, amorphous structure that is more susceptible to plastic deformation under stress [102]. With the introduction of PLA and its increasing ratio, the rigid molecular chains interfere with the cohesive, deformable network of Fb molecules, limiting the molecular mobility required for significant plastic flow and energy dissipation prior to fracture [72]. The formation of crystalline domains in PLA enhances tensile strength but also creates barriers that restrict chain slippage and reorientation, limiting the fiber’s overall strain capacity [27].

The NFs matched the pH of the buccal mucosa (typically pH 6.2–7.4), which is essential for patient comfort and safety, preventing irritation or tissue damage caused by significant pH deviations [107]. This could indicate their compatibility with the oral mucosa and non-irritancy as well.

The FTIR peaks of EMPA in CS/Fb/PLA NFs underscore EMPA’s unique contributions to the scaffold’s composition. These peaks suggest that the successful integration of EMPA into NFs enhanced physical interactions and synergistic effects among the components, ultimately improving the scaffolds’ mechanical and biological properties.

The XRD for the optimized composite scaffold F8 demonstrated lower crystallinity and enhanced amorphous characteristics compared to the EMPA powder. These results suggest a high degree of uniformity among the compositions.

The initial burst drug release is a common drawback of the hydrophilic polymer-based NFs, which originates from the adsorbed drug on the scaffold surfaces. However, tuning of NF composition via blending with hydrophobic polymers could modulate the release pattern of the loaded drug into a more sustained one [108].

Applying the best fit, the release model showed a Korsmeyer–Peppas model with a low release exponent (n < 0.45), which suggests a pseudo-Fickian diffusion mechanism, wherein drug release is predominantly influenced by Fickian diffusion, albeit with supplementary limitations, including polymer-drug interactions or matrix constraints that impede the release rate like swelling or erosion [109]. This is consistent with findings on sertaconazole-loaded FB-based NFs, where a comparable low n value (0.19) was reported [110]. A Weibull β value lower than 1 implies an exponential decay in the release rate over time, consistent with diffusion-dominated systems where a readily accessible drug near the fiber surface is released quickly, followed by slower diffusion of the drug from the inner core of the nanofiber. This phenomenon may result from the gradual erosion or swelling of the polymer. Multi-phase kinetics have been observed in PLGA-based systems, where a β value greater than 1 indicates degradation-controlled release patterns. The comparatively lower R2 of the Higuchi model suggests that while diffusion is a principal factor, the release kinetics might be influenced by additional factors such as polymer-drug interactions or matrix heterogeneity, which the simpler Higuchi model does not fully capture [110].

The results align with a previous investigation of PLA/ibuprofen nanofibers which demonstrate a similar diffusion-restricted release (n ≈ 0.25–0.30) in physiological conditions, which can be ascribed to the interactions between the polymer and the drug [108,111]. The intricate mechanisms observed in polymeric nanofibers exemplify how the interplay of material composition and structural design influences the kinetics of release.

Despite extensive research, the complex molecular mechanisms driving AD progression remain incompletely understood. Among the proposed mechanisms, the pathological interplay between phosphorylated tau (p-tau), amyloid-β (Aβ), and the receptor for advanced glycation end-products (AGER) plays a critical role [112]. Aβ interacts with AGER on neurons, microglia, and endothelial cells, initiating intracellular signaling cascades that activate nuclear factor-kappa B (NF-κB), and upregulate pro-inflammatory cytokines [113]. This inflammatory milieu promotes the activation of tau kinases, leading to abnormal tau phosphorylation and the accumulation of neurofibrillary tangles [114]. Concurrently, p-tau exacerbates Aβ toxicity by impairing cellular homeostasis and facilitating mitochondrial dysfunction [113]. The persistent activation of glial cells through AGER signaling sustains neuroinflammation, further promoting Aβ production and tau pathology, thus establishing a self-reinforcing cycle of neurodegeneration [115]. Targeting the Aβ–AGER–p-tau axis presents a promising therapeutic strategy for disrupting this deleterious cascade in AD.

Aluminum chloride (AlCl_3_) exposure in rats has been widely utilized to model AD because of its neurotoxic effects that replicate essential features of the human situation [116]. Research by Khan et al. (2024) demonstrated that the treatment of AlCl_3_ results in considerable cognitive deficits and neurodegenerative alterations [117]. Oral administration of AlCl_3_ (100 mg/kg) for 21 days led to a substantial elevation of Aβ and p-tau concentrations. The oral administration of AlCl_3_ significantly elevated the RNA expression of AGE receptors in brain tissues. Alqarni et al. (2024) documented similar findings, indicating that AlCl_3_ has prompted the accumulation of amyloid-beta (Aβ) peptides and hyperphosphorylated tau proteins, both of which are characteristic hallmarks of AD [118]. The precise function of the receptor for advanced glycation end-products (AGER) in this context remains ambiguous; however, the cumulative data indicates that AlCl_3_ interferes with essential processes implicated in AD pathogenesis, such as Aβ accumulation and tau hyperphosphorylation [119]. Furthermore, Fanlo-Ucar et al. (2024) reported that AlCl_3_ has induced an increase in pro-inflammatory cytokines, leading to neuronal injury [120]. Consistent with this finding, our results demonstrated a significant elevation in IL-1β following oral administration of AlCl_3_. These findings highlight the efficacy of AlCl_3_-induced models in examining the intricate pathways associated with AD [121].

Memantine is a non-competitive antagonist of the N-methyl-D-aspartate (NMDA) receptor, approved for the management of moderate to severe AD [122]. It inhibits calcium overload and reduces excitotoxic neuronal damage, a characteristic feature of AD pathology [53]. In addition to its neuroprotective properties, preclinical studies indicate that memantine may reduce amyloid beta (Aβ)-induced neurotoxicity by inhibiting Aβ-induced NMDA activation and calcium influx, which could affect Aβ aggregation and clearance [123]. The present study corroborates this finding, demonstrating that oral administration of memantine (5 mg/kg) for 21 days alongside AlCl_3_ significantly reduced Aβ, p-tau, IL-1β, and also attenuated AGE receptor expression, with significant decreases as compared to the positive control group. Research indicated that memantine reduced tau hyperphosphorylation, likely by inhibiting tau kinases such as GSK-3β, which in turn decreased the formation of neurofibrillary tangles [124]. Memantine does not directly antagonize the receptor for advanced glycation end-products (AGER), but it may indirectly downregulate AGER-mediated signaling pathways by reducing oxidative stress and neuroinflammation, which are both exacerbated by AGER activation in AD [125].

In recent years, there has been growing interest in the potential role of empagliflozin in the treatment of AD, particularly in animal models. While the exact mechanisms of empagliflozin in AD are still being investigated, early studies in rats and preclinical models indicate that this drug could have a significant role in modulating disease-related pathways, offering hope for potential therapeutic applications in AD [126]. In rats, studies have shown that SGLT2 inhibitors, including empagliflozin, may offer neuroprotective effects beyond their glucose-lowering properties. In the current study, we have declared that administration of EMPA-Cs/Fb/PLA-NFs concurrently with AlCl_3_ has exerted a significant protective role against AD, which was demonstrated by the significant amelioration of Aβ, p-tau, and IL-1β concentration, and also the attenuation of AGE receptor expression in brain tissue with significant decreases as compared to the positive control group and the plain Cs/Fb/PLA-NFs group.

One of the proposed mechanisms is that empagliflozin may reduce oxidative stress and inflammation, both of which are key contributors to the progression of AD [127]. Additionally, empagliflozin has been found to reduce amyloid-beta accumulation and tau phosphorylation, two pathological hallmarks of AD, by influencing metabolic and inflammatory pathways in the brain [128]. The multifaceted actions indicate that empagliflozin may have broader neuroprotective effects beyond those of SGLT2 inhibitors. The novel nanoform of empagliflozin (EMPA-Cs/Fb/PLA-NFs) demonstrated a notable protective effect compared to pure EMPA.

AlCl_3_ exposure has been shown to impair motor function and reduce the exploratory behavior of rats, which correlates with the development of memory and learning deficits seen in AD [129]. The present investigation demonstrated that oral administration of AlCl_3_ (100 mg/kg) for 21 days to the positive control group led to a substantial decrease in spontaneous locomotor activity of the rats, compared to the negative control group. The results also indicated no significant difference between plain Cs/Fb/PLA-NFs and the positive control group. Administration of memantine (5 mg/kg) concurrently with AlCl_3_ for 21 days led to a significant increase in spontaneous locomotor activity as compared to the positive control group. It was reported that memantine may enhance locomotor activity by modulating glutamatergic neurotransmission, thus improving cognitive functions and motivation that can lead to increased spontaneous movement [130,131].

In AlCl_3_-induced AD models, the T-maze has been used to evaluate cognitive deficits, particularly memory retrieval and learning. AlCl_3_ administration has been found to impair performance on the T-maze task, as rats exposed to AlCl_3_ have difficulty learning or recalling the correct arm, a behavior indicative of spatial memory impairment and hippocampal dysfunction [132]. In the current study, oral administration of AlCl_3_ resulted in a significant decrease in the alternation of rats in the T-maze test as compared to the negative control group. Similar results were reported by El-Kashak et al. (2025) [57]. Empagliflozin may enhance cognitive function as well as locomotor activity by addressing the inflammatory response and key pathological markers of AD (Aβ and p-tau), hence augmenting spontaneous locomotor activity in animal models of AD [126].

## 5. Conclusions

EMPA-loaded Cs/Fb/PLA nanofibers were fabricated by electrospinning to evaluate their anti-Alzheimer’s potential via buccal delivery. SEM analysis confirmed uniform, bead-free fibers, while FTIR and XRD results verified successful drug incorporation, molecular interactions with the polymer matrix, and reduced crystallinity of EMPA. The optimized formulation exhibited favorable swelling behavior, hydrophilicity, high drug content, and a biphasic sustained release profile. In vivo, EMPA-Cs/Fb/PLA nanofibers improved cognitive and locomotor performance, reduced pathological markers associated with AD, and provided neuroprotection comparable to standard therapy. Histopathological and immunohistochemical assessments further supported preservation of the hippocampal neuronal structure and restoration of synaptic integrity. According to these findings, buccal administration of EMPA via Cs/Fb/PLA nanofibers represents a novel, non-invasive, and sustained-release therapeutic strategy for AD management, combining drug repurposing with advanced nanofiber-based delivery to achieve multimodal neuroprotection.

## Figures and Tables

**Table 1:** The concentrations of different constituents incorporated in the fabrication of the blank and EMPA-loaded nanofibrous scaffolds.

Constituents	Investigated Ranges			
**Fb concentration**	15%			
**PLA concentration**	15%			
**Fb:PLA (** *v* **/** *v* **)**	1:0	2:1	1:1	1:2
**Cs concentration (%** *w* **/** *v* **)**	0%		1%	
**EPMA concentration (%** *w* **/** *v* **)**	0%	1%	3%	5%

Fb: fibroin; Cs: chitosan; PLA: polylactic acid; and EMPA: empagliflozin.

**Table 2:** Primer’s sequence of all studied genes.

Gene Symbol	Primer Sequence from 5′-3′
**AGE receptor**	F: AGC TTC AGT CTG GGC CTT C R: CAG CTG AAT GCC CTC TGG L33413.1
**GAPDH**	ATGGTGAAGGTCGGTGTGAACG TGGTGAAGACGCCAGTAGACTC NM_001411843.1

**Table 3:** Electrospinning parameters of different nanofiber formulations.

Scaffolds’ Code	Fb:PLA (*v*/*v*)	Fb conc. (%*w*/*v*).	PLA conc. (%*w*/*v*)	Cs conc. (%*w*/*v*)	EMPA (*w*/*v*)	Voltage (Kv)	Feed Rate (mL/h)	Average Fiber Diameter (AFD) (nm ± S.D)	Physical Observation
**F1**	1: 0	15%	15%	0%	-	24	0.5	350 ± 129	Fibers with beads
**F2**	1: 1	15%	15%	0%	-	28.5	0.9	290 ± 236	Good fibers
**F3**	1: 2	15%	15%	0%	-	28	0.6	239 ± 176	Noncontinuous fibers
**F4**	2:1	15%	15%	0%	-	28.5	0.4	227 ± 96	Smooth continued fibers
**F5**	2:1	15%	15%	1%	-	30	0.3	175 ± 43	Good fibers with little beads
**F6**	2:1	15%	15%	1%	1%	30	0.5	668 ± 148	Very good fibers
**F7**	2:1	15%	15%	1%	3%	30	0.6	459 ± 173	Bead-less fibers
**F8**	2:1	15%	15%	1%	5%	30	0.5	526 ± 177	Stretchable smooth fibers

**Table 4:** Mechanical properties and drug content of pure Fb, Fb/PLA, and Cs/Fb/PLA electrospun nanofibers.

Formula Code	Moisture Loss (%)	Tensile Strength (N/cm^2^)	Elongation-at-Break (%)	Drug Content (% *w*/*w*)	Bulk Ph
**F1**	3.57 ± 0.91	0.83 ± 0.03	49.66 ± 3.5	__	6.7 ± 0.06
**F2**	8.11 ± 0.76	2.26 ± 0.14	19.04 ± 2.6	__	7.07 ± 0.05
**F3**	19.23 ± 0.62	2.98 ± 0.17	11.25 ± 3.4	__	7.06 ± 0.06
**F4**	4.08 ± 0.82	1.89 ± 0.18	12.16 ± 0.9	__	7.01 ± 0.03
**F5**	12.5 ± 0.74	4.32 ± 0.21	13.72 ± 1.1	__	7.11 ± 0.05
**F6**	5.17 ± 0.92	3.20 ± 0.19	12.62 ± 1.9	2.3 ± 0.9	7.03 ± 0.04
**F7**	4.01 ± 0.75	2.15 ± 0.09	12.86 ± 2.1	6.7 ± 0.8	7.04 ± 0.08
**F8**	1.69 ± 0.69	1.24 ± 0.16	11.18 ± 1.6	12 ± 1.7	6.99 ± 0.03

## Data Availability

The data underpinning the outcomes of this investigation can be obtained from the corresponding author following a reasonable inquiry.

## Supplementary Materials

The following supporting information can be downloaded at: https://www.mdpi.com/article/10.3390/pharmaceutics18010083/s1. Table S1: Experimental Design and Treatment Groups. Figure S1: kinetics modeling of the optimized formulation to different kinetic models. R2: correlation coefficient, n = diffusion coefficient, β: shape parameter.
