# Supplementary material for: Novel Repurposing of Empagliflozin-Loaded Buccal Composite (Chitosan/Silk Fibroin/Poly(lactic acid)) Nanofibers for Alzheimer’s Disease Management via Modulation of Aβ–AGER–p-tau Pathway
Source: Pharmaceutics. 2026 Jan 8;18(1):83. doi: 10.3390/pharmaceutics18010083 (PMC12844634; doi:10.3390/pharmaceutics18010083)
Supplement: Supplementary file 1 [file pharmaceutics-18-00083-s001.zip › pharmaceutics-4060826-supplementary.pdf]

Supplementary data

Table S1: Experimental Design and Treatment Groups

Group	Description	Treatment	Dose	Duration	Notes
I	Negative Control	Saline	1 ml/kg	21 days	—
II	Positive Control	Aluminum chloride ($AlCl_3$)	100 mg/kg	21 days	—
III	EMPA (Pure)	EMPA	20 mg/kg	21 days	Given concurrently with $AlCl_3$
IV	Standard Drug	Memantine	5 mg/kg	21 days	Given concurrently with $AlCl_3$
V	Plain Group	Plain Cs/Fb/PLA-NFs (buccal films)	—	21 days	Given concurrently with $AlCl_3$
VI	EMPA-NFs Group	EMPA-Cs/Fb/PLA-NFs (buccal films)	20 mg/kg	21 days	Given concurrently with $AlCl_3$

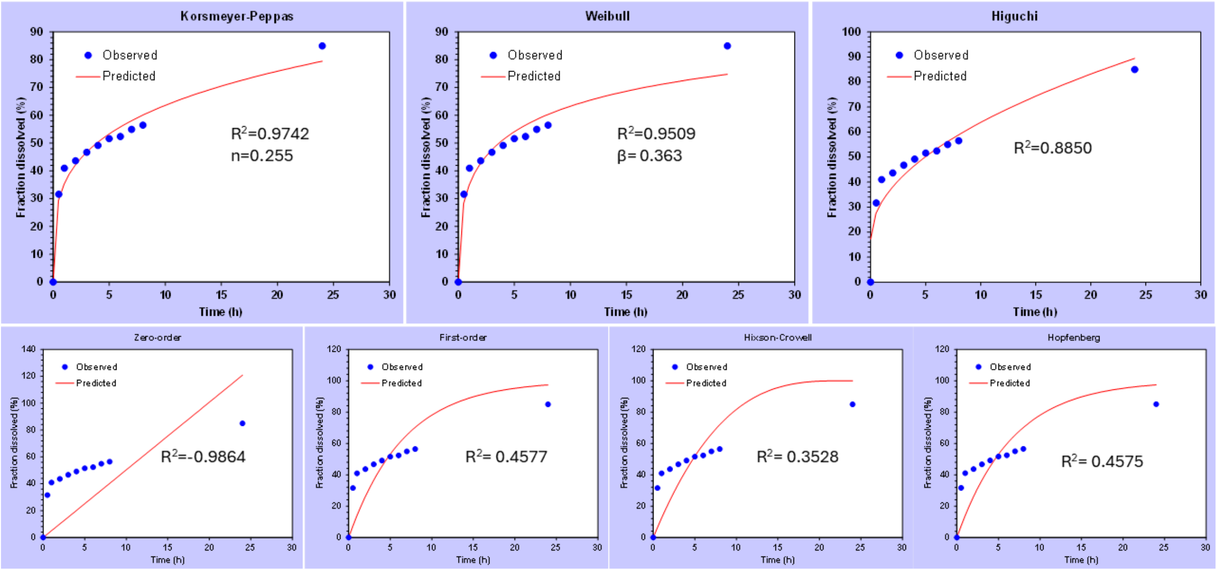

Figure S1: kinetics modeling of the optimized formulation to different kinetic models. R2: correlation coefficient, n= diffusion coefficient, β: shape parameter.
